# Non-response to first-line hormonal treatment for symptomatic endometriosis: overcoming tunnel vision. A narrative review

**DOI:** 10.1186/s12905-023-02490-1

**Published:** 2023-06-30

**Authors:** Giulia Emily Cetera, Camilla Erminia Maria Merli, Federica Facchin, Paola Viganò, Elisa Pesce, Francesca Caprara, Paolo Vercellini

**Affiliations:** 1 Gynecology Unit, Fondazione IRCCS Ca’ Granda Ospedale Maggiore Policlinico, Milan, Italy; 2 Department of Psychology, Catholic University of the Sacred Heart, Milan, Italy; 3 Infertility Unit, Fondazione IRCCS Ca’ Granda Ospedale Maggiore Policlinico, Milan, Italy; 4 Department of Clinical Sciences and Community Health, University of Milan, Milan, Italy

**Keywords:** Endometriosis, Progesterone-resistance, Non-response, Pain contributors, Central sensitization, Myofascial pain, Mind-body framework

## Abstract

One-fourth to one-third of women with endometriosis receiving first-line hormonal treatment lacks an adequate response in terms of resolution of painful symptoms. This phenomenon has been ascribed to “progesterone resistance”, an entity that was theorized to explain the gap between the ubiquity of retrograde menstruation and the 10% prevalence of endometriosis among women of reproductive age.

Nevertheless, the hypothesis of progesterone resistance is not free of controversies. As our understanding of endometriosis is increasing, authors are starting to set aside the traditionally accepted tunnel vision of endometriosis as a strictly pelvic disease, opening to a more comprehensive perspective of the condition. The question is: are patients not responding to first-line treatment because they have an altered signaling pathway for such treatment, or have we been overlooking a series of other pain contributors which may not be resolved by hormonal therapy?

Finding an answer to this question is evermore impelling, for two reasons mainly. Firstly, because not recognizing the presence of further pain contributors adds a delay in treatment to the already existing delay in diagnosis of endometriosis. This may lead to chronicity of the untreated pain contributors as well as causing adverse consequences on quality of life and psychological health. Secondly, misinterpreting the consequences of untreated pain contributors as a non-response to standard first-line treatment may imply the adoption of second-line medical therapies or of surgery, which may entail non-negligible side effects and may not be free of physical, psychological and socioeconomic repercussions.

The current narrative review aims at providing an overview of all the possible pain contributors in endometriosis, ranging from those strictly organic to those with a greater neuro-psychological component. Including these aspects in a broader psychobiological approach may provide useful suggestions for treating those patients who report persistent pain symptoms despite receiving first-line hormonal medical treatment.

## Background

It has been estimated that one-fourth to one-third of women with endometriosis receiving combined oral contraceptives (COCs) or progestins lack an adequate response to treatment in terms of resolution of painful symptoms [1]. This phenomenon has been ascribed to “progesterone resistance”, an entity that was theorized at the beginning of the third millennium to explain the gap between the ubiquity of retrograde menstruation and the 10% prevalence of endometriosis among women of reproductive age [2, 3].

Moving from the hypothesis that women who develop endometriosis may do so due to an abnormal endometrium, authors started conducting molecular studies both on the eutopic and ectopic endometrium of these patients, obtaining conflicting results. A constant of many studies, however, was the finding of a reduced expression of progesterone receptors PR-A, and especially PR-B, both in the ectopic endometrium [3–7] and in the eutopic endometrium [8, 9] of affected patients. In particular, the reduced expression of PR-A and PR-B, which may be responsible for an enhanced proliferation of endometrial cells, was ascribed by Wu and co-workers to the hypermethylation of the progesterone receptor promoter, caused by persistent inflammation [6]. Bulun’s group, however, speculated that a deficient methylation of the estrogen receptor ERβ promoter might be involved [4].

Moreover, studies on embrio-implantation in women with endometriosis reported an attenuated decidualization and a downregulation of various progesterone target genes during the implantation window [8–11].

This body of evidence may not only explain the enhanced proliferative property of endometrial cells in patients with endometriosis, and consequently the gap between retrograde menstruation and disease development. It may also partly justify the effect of endometriosis on fertility, as well as its variable response to treatment. For this reason, so-called “progesterone resistance” has been adopted in the last decades both as a pathogenic theory and as an explanation for refractoriness to progestin therapy in terms of persistence of painful symptoms, i.e., dysmenorrhea, noncyclical pelvic pain, dyspareunia, dysuria and dyschezia.

Nevertheless, the hypothesis of progesterone resistance is not free of controversies. Both Bukulmez and Gentilini’s groups, for example, failed to prove a reduced expression of progesterone receptors in the endometrium of affected patients [12, 13]. Most importantly, as our understanding of endometriosis as a chronic, multifactorial, inflammatory process with a systemic nature is increasing [14], authors are starting to set aside the traditionally accepted tunnel vision of endometriosis as an estrogen-dependent and strictly pelvic disease, opening to a more comprehensive perspective of the condition, including physical and mental health [15]. The central question is: are patients not responding to standard treatment because they have an altered signaling pathway for such treatment, or have we been overlooking a series of other pain contributors which may not be resolved by hormonal therapy?

Finding an answer to this question is evermore impelling, for two reasons mainly. Firstly, because not recognizing the presence of further pain contributors adds a delay in treatment to the already existing delay in diagnosis of endometriosis, that consists on average of seven to 12 years from the onset of symptoms [14]. This delay may exacerbate the untreated pain contributors, leading to their chronicity, as well as causing adverse consequences on quality of life, psychological health, intimate relationships and daily activities [16–20]. Secondly, misinterpreting the consequences of untreated pain contributors as a non-response to standard first-line treatment may imply the adoption of second-line medical therapies or of surgery, which may entail non-negligible side effects and may not be free of physical, psychological and socioeconomic repercussions.

The current narrative review aims at providing an overview of all the possible pain contributors in endometriosis, ranging from those strictly organic to those with a greater neuro-psychological component. Including these aspects in a broader psychobiological approach may provide useful suggestions for treating those patients who report persistent pain symptoms despite receiving first-line hormonal medical treatment.

## Brief overview of possible pain contributors in endometriosis

Pain is a complex perception, which results from the interaction between peripheral sensory inputs, their central processing, cortical activation and, finally, behavioral response [21, 22]. Briefly, nociceptive signals are conveyed from the periphery to several thalamic nuclei along the spinothalamic tract and are subsequently projected to the cortex. Multiple cortical areas are activated simultaneously and communicate with subcortical structures, in order to provide different aspects of the pain experience, such as spatial and temporal characterization, conscious perception, emotional valence, modulation of pain magnitude and cognitive elaboration [23, 24].

Persistent pain occurs when the perception of pain does not abate despite its causative agent has been eliminated [22]. This may occur due to a dysfunction in several locations of the pain pathways simultaneously. The lowering of the threshold of peripheral sensory nociceptors, which consequently respond to liminal and subliminal inputs to a greater extent, is named “peripheral sensitization” (PS). Conversely, “central sensitization” (CS) may occur when a defect in the synapses of the spinal cord and of more rostral areas including the brainstem, the thalamus and the cortex amplifies pain perception [21, 25].

**Fig. 1:**
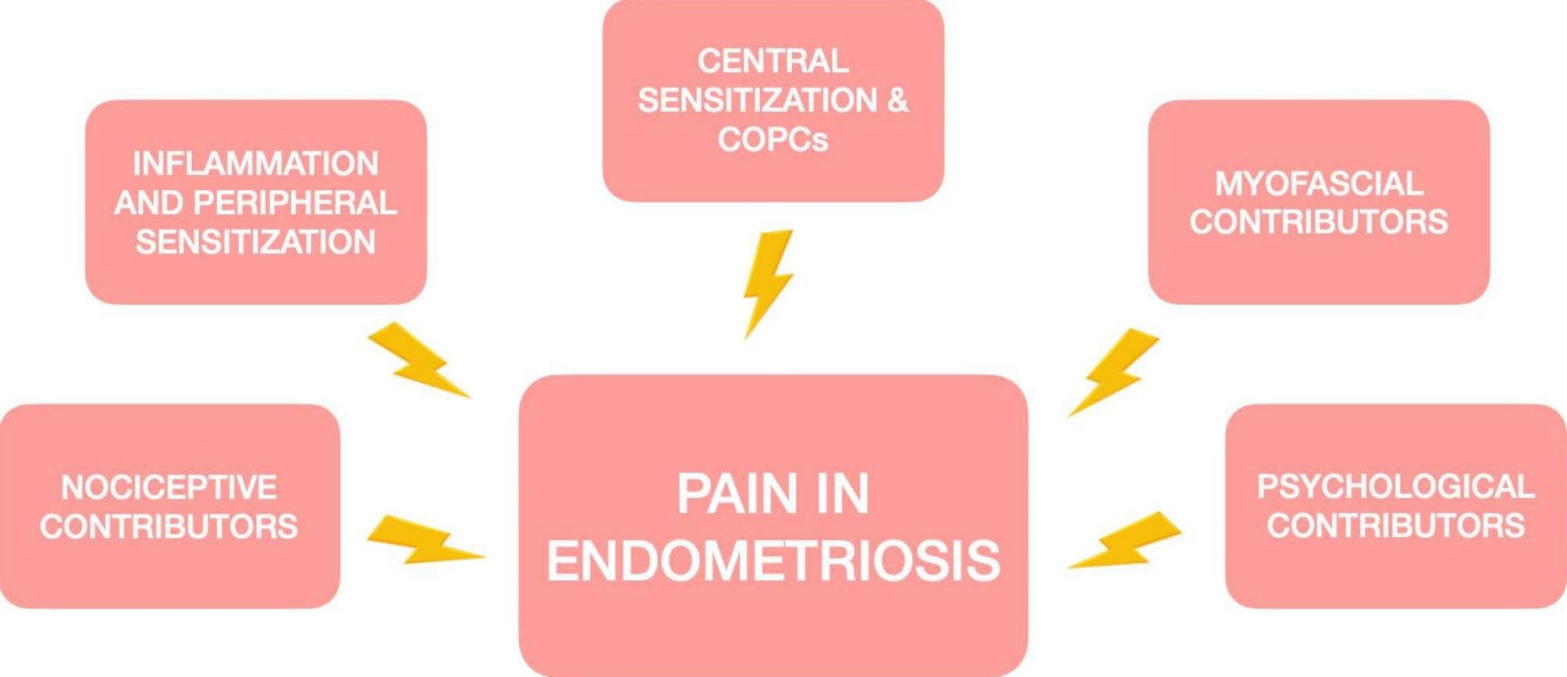
Pain contributors in endometriosis

Given these premises, the shift towards the inclusion of endometriosis-related persistent pain in a broader framework, which considers both peripheral and central contributors to pain, appears inevitable. In these regards, Ezra and co-workers have recently classified mind-body interrelationships in four clusters, in which the mind-body ratio is progressively increasing (cluster 1: organic conditions; cluster 2: stress-exacerbated, typically inflammatory diseases; cluster 3: functional somatic syndromes; cluster 4: conversion disorders) [26]. Owing to its organic, inflammatory, multifaceted nature, endometriosis may be situated in all the first three clusters (the fourth cluster must be categorically excluded when defining endometriosis). As such, its symptoms may be the result of the interaction of numerous contributors (Fig. 1), which we briefly overview.

### Nociceptive contributors

Nociception occurs via the direct activation of peripheral pain receptors, i.e. nociceptors, which evoke excitatory currents that are conveyed to the central nervous system [27]. Over the past years, ample evidence regarding the presence of myelinated nerve fibers and of nociceptors in or near endometriotic lesions has been collected. These nociceptors are activated by the release of algogens, including inflammatory molecules, from the ectopic endometrium in the peritoneal fluid (PF) [28–30].

In cases of deep infiltrating endometriosis, the swelling of the foci entrapped in fibrotic tissue, the infiltration of visceral walls and the mechanical stimulation of scar tissue and adhesions may concur to the development of pain [29, 31, 32].

### Inflammation and peripheral sensitization

It is not yet known if inflammation instigates or perpetuates endometriosis. What is certain is that it is an essential feature of the disease, as the presence of a widespread inflammatory environment has been proven inside and outside the pelvis of affected patients [33].

Suryawanshi and co-workers reported that endometriotic lesions possess a specific immune microenvironment, which resembles a tumor inflammatory profile [34]. In particular, pro-inflammatory cytochines such as interleukin (IL)-1β, IL-6, IL-8 and nerve growth factor (NGF) have been found increased in the PF and within endometriotic lesions [35], while prostaglandins E2 are overexpressed within the uterine endothelial cells, due their estradiol-dependent production [36]. Also, immune cell populations appear to be altered in these patients. Neutrophil granulocytes are recruited in the PF in greater concentrations [37], as are lymphocytes [38–40]. Macrophages, on the other hand, display reduced phagocytic capacity compared to healthy controls [39, 41–43].

Causes underlying the development of chronic inflammation in endometriosis are yet to be fully understood, although debris produced by retrograde menstrual blood flow seem to be involved [44]. However, the role of so-called peripheral neuroinflammation, i.e. the vicious circle by which peripheral nerve endings secrete pro-inflammatory neuromodulators in response to their infiltration by macrophages, is gaining increasing attention [43, 45]. Recent evidence supports the hypothesis that neuroinflammation may be the underlying cause of PS, the process by which defective peripheral sensory nociceptors cause hyperalgesia, an exaggerated perception of painful stimuli [43]. Moreover, an imbalance between an increased density of sensory nerve fibers, which release pro-inflammatory transmitters, and a decreased density of sympathetic nerves, which may induce an anti-inflammatory effect, has been found in endometriotic lesions [46, 47].

Therefore, inflammation and pain appear to be tightly intertwined in a mechanism by which one maintains and aggravates the other, thus causing PS [27, 48].

### Central sensitization and chronic overlapping pain conditions (COPCs)

Central sensitization is a physio-pathological process whereby a patient becomes more sensitive to peripheral stimuli via central neural mechanisms, which are similar to those underlying the generation of memory [49].

The *primum movens* of CS is not fully known. However, it has been hypothesized that a continued peripheral input, such as inflammation, may induce sensory neurons in the dorsal horn of the spinal cord to respond at a higher frequency to nociceptive and non-nociceptive inputs. This may cause hyperalgesia, allodynia, persistence of pain perception even when the noxious input has been eliminated and an increase in the receptive field size [21, 26]. A poor functioning of more rostral structures such as the periaqueductal gray area, which is deputed to endogenous analgesia, has also been described in patients with CS [49, 50]. These individuals, in fact, show an increased sensitivity to experimental nociceptive stimuli also in areas of the body not related to the primary disease [51]. Lastly, CS is often accompanied by psychological responses such as catastrophic misinterpretation, selective attention and fear-based conditioning, and by constitutional symptoms such as sleep disturbances, cognitive dysfunction and asthenia [26, 51].

The role of CS in the development and in the perception of endometriosis-related pain is being increasingly reported by the literature [14, 21]. Not only, endometriosis has been included in the National Institutes of Health Pain Consortium list of Chronic Overlapping Pain Conditions (COPCs), a set of chronic pain conditions which often co-occur, appear to share CS as a common underlying mechanism and are often associated with mood disorders [51].

Interestingly, studies on women suffering from endometriosis report that those with a greater central component to pain are less responsive to treatment [52, 53]. In their study, Raimondo and co-workers reported a 41.4% prevalence of CS among 285 consecutive women with endometriosis. Moderate to severe pain symptoms, except for dyschezia, were significantly more frequent in the CS group and the rate of failure of first-line hormonal treatment was greater among these patients compared to the non-CS group [52]. Similarly, Orr and colleagues found more severe ratings of pain, an earlier onset of pain and a greater probability of non-response to hormonal therapy among women with endometriosis and signs of CS [53].

### Myofascial contributors

The musculoskeletal system is often overlooked in the evaluation of chronic pelvic pain, mainly due to the fact that care providers don’t feel adequately knowledgeable in this regard [54]. However, it has been extensively proven that the central elaboration of repeated peripheral nociceptive inputs may induce the development of viscero-somatic reflexes which result in an increased muscle tone in the pain-related area [55]. The contracture of the muscle tissue and of its related fascia may lead to the generation of myofascial trigger points, hard, palpable nodules which are painful upon compression. Trigger point-associated pain may be due both to a high local concentration of inflammatory algogens and to muscle hypoxia and acidosis due to prolonged muscle contraction [54].

According to Till and co-workers, myofascial contribution to pain may be recognized in as many as 60–90% of women with chronic pelvic pain, including those with endometriosis [51]. These patients typically report hypertonus-related pain as non-cyclic soreness, cramping, stabbing or throbbing in the lower abdomen, often described as “ovarian pain”. Pain may also radiate to pelvic organs such as the vagina, the vulva, the bladder or the rectum, and to musculoskeletal districts such as the hips, the buttocks or the lower limbs. Dyspareunia may be a further expression of myofascial pain [51, 54]. In a recent study on 30 women with endometriosis, although 77% were using hormonal treatment, 97% reported non-menstrual pelvic pain. All participants were found to have pelvic floor spasm and myofascial trigger points and all acknowledged the pelvic floor as a major focus of their pelvic pain [56].

### Psychological contributors

The triad anxiety, depression and fatigue is a common trait of many chronic inflammatory diseases, including endometriosis [15, 39, 57]. Further mental health comorbidities include bipolar disorder (OR 6), alcohol/drug dependency (3.5%), eating disorders (1.5-9%) and hyperactivity disorder (4%) [58–61]. Early psychological and physical trauma has also been found in these patients [62].

Impaired psychological health in individuals with endometriosis has been traditionally related to the presence of pain symptoms, especially chronic pelvic pain, and to the overall psychosocial burden of living with the disease. However, recent studies provided evidence of a more complex interaction among multiple factors – including inflammation, central sensitization, hormonal treatment, genetic predisposition, and the overall impact of endometriosis, especially when symptomatic – that may explain the prevalence of psychological symptoms in this population [15, 58, 63].

As the understanding of mind-body interrelations increases, the connection between pain and psychological health in a vicious-circle manner is becoming progressively evident. Psychological distress and dysfunctional pain management, including catastrophizing, may in fact increase the perception of pain by acting on central synapses and by inducing hypertonus of the pelvic myofascial structures, which are particularly vulnerable to psychological stress [27, 64]. At the same time, dealing with endometriosis-related pain remains an important cause of psychological suffering [27].

## Management of pain contributors in endometriosis

Although brief, our overview aims to highlight the necessity of an open-minded, comprehensive and multidisciplinary approach when treating patients affected by endometriosis. In fact, we agree with Till and co-workers that the optimal management of chronic pain conditions must address all peripheral and central contributors [51]. Failing to diagnose and treat, if necessary, all possible etiologic factors may lead physicians on the slippery slope of believing hormonal treatment is not effective, when it may actually be necessary but not sufficient to treat patients exhaustively.

### Treatment of nociceptive pain

This form of pain is generally well managed with first-line hormonal treatment (COCs or progestins) and non-steroidal anti-inflammatory drugs (NSAIDs). While the former arrest ovulation, and as such the cycle-related release of algogens, the latter inhibit cyclooxygenase, further decreasing the levels of prostaglandins [53]. In cases of deep lesions, symptoms are controlled by first-line hormonal treatment in about two thirds of patients. Progestins in fact induce atrophy of the ectopic endometrium, contrasting its infiltration of pelvic organs [65].

### Reduction of inflammation

Targeting deregulated immune pathways may represent a potential avenue for novel therapeutic strategies in endometriosis [39, 44]. Currently, however, no such treatment is available for clinical use.

Various authors have studied the effectiveness of regular physical activity and of anti-inflammatory diets as a way to reduce inflammation, and thus improve painful symptoms.

Regular physical exercise appears to increase systemic levels of anti-inflammatory cytokines in patients with chronic inflammatory diseases [66]. In women with endometriosis, exercise may further prove beneficial as it increases sex hormone-binding globulin levels, thus reducing estrogen levels [67]. Despite such evidence, in their systematic review, Hansen and colleagues failed to prove any beneficial effect of exercise on pain perception in women with endometriosis [68]. However, the six studies included in the review were based on low quality, heterogeneous data and were conducted on small cohorts of women.

Regarding dietary interventions, in 2022 Nirginakis and co-workers investigated their effect on endometriosis-related painful symptoms by conducting a systematic review of the literature. Results included weak evidences regarding possible advantages of a Mediterranean diet; antioxidant supplementation with vitamins (B6, A, C, E), mineral salts (Ca, Mg, Se, Zn, Fe), lactic ferments, fish oil (omega-3/6); a gluten-free diet and a low intake of fermentable oligo-, di-, monosaccharides, and polyols (FODMAP diet) - the latter was analyzed in a population of women suffering both from endometriosis and irritable bowel syndrome -.

Mediterranean diet, among all, has well-known antioxidant effects. In particular, extra virgin olive oil displays a similar structure to the molecule ibuprofen, and as such is able to inhibit cyclooxygenase. This considered, although evidence regarding its efficacy on symptom relief is scarce, the authors concluded that clinicians may suggest this type of diet to patients with endometriosis as a long-term dietary change [66].

### Treatment of central sensitization

It has been hypothesized that the contribution of CS to pain perception is not comparable in all patients suffering from the same chronic pain condition 69. For this reason, two self-reported questionnaires have been created and validated to aid physicians in the assessment of CS-related symptoms in clinical practice. These include the Central Sensitization Inventory (CSI), a 25-item questionnaire which investigates both CS-related symptoms and the presence of other COPCs (scores ≥ 40 are indicative of CS) 69; and the 2011 Fibromyalgia Survey Score, which analyzes the total number of painful areas on a body map and the severity of such pain. The latter questionnaire may be used to diagnose both fibromyalgia and the degree of CS in other chronic pain conditions [62].

Various pharmacologic and non-pharmacologic treatments have been suggested to manage CS, often with modest or conflicting results, mainly due to the fact that the mechanisms behind CS are not fully understood yet.

The limited evidence regarding pharmacological treatments includes studies on antidepressants, centrally acting muscle relaxants, antiepileptic drugs and cannabinoids.

Tricyclic antidepressants (TCAs) and serotonin–norepinephrine reuptake inhibitors (SNRIs) are the most commonly used antidepressants for the treatment of chronic pain conditions. Their efficacy in decreasing pain sensitivity is mediated by the inhibition of norepinephrine reuptake in the descending pain modulatory pathways. TCAs may cause bothersome side effects more frequently compared to SNRIs and are associated with less robust improvement [70, 71]. Interestingly, in a recent overview of 26 systematic reviews, Ferreira and co-workers failed to find high certainty evidence regarding the effectiveness of antidepressants for chronic pain conditions, raising the question whether they should be routinely prescribed in these patients [72].

Centrally acting muscle relaxants, such as cyclobenzaprine, also inhibit norepinephrine uptake, and may play a role in reducing the hypertonus of pelvic muscles [51].

The prescription of gabapentinoids in the treatment of CS is debated. These centrally acting calcium channel blockers are typically used in the treatment of epilepsy but are also extensively used in neuropathic pain conditions as they decrease activity in the ascending pain pathways, as well as having some membrane stabilization activity [51]. Their efficacy in chronic pelvic pain appears to be limited, probably due to the fact that neuropathic pain is not the main mechanism in the etiopathogenesis of this condition. In their randomized, placebo-controlled trial, Horne and colleagues failed to find a significant reduction in pain scores among patients receiving Gabapentin, in spite of an increased risk of drug-related side effects, as compared to placebo [73].

In the intent of overcoming the flaw in medical treatment of CS, researchers have analyzed the possible therapeutic role of cannabinoids. However, evidence regarding their efficacy or safety is still limited [74–76].

As what regards evidence on non-pharmacological treatments of CS, this is mainly low quality and includes studies on physical exercise, psychotherapy and acupuncture.

Physical exercise has been shown to improve pain, mood and sleep quality in patients suffering from chronic pain conditions. Aerobic exercise, resistance and yoga seem to be equally effective, although the reason why they are effective is still unknown. Probably their anti-inflammatory effect, the boosting of psychological well-being and of sociality, the improvement in muscle function and the increased pain tolerance due to repeated exposure to low levels of exercise-related discomfort play a role [14, 66].

In the endometriosis population, several forms of psychological interventions – such as psychological counseling and support – may help people identify a more effective and personalized strategy to manage the disease, especially in cases of negative pain management characterized by dysfunctional coping strategies, catastrophic thinking, and high levels of anxiety, which may also lead to avoidant behaviors and isolation. As regards psychotherapy, the extant endometriosis research focused on cognitive behavioral therapy (CBT) and provided evidence that it may be effective in the context of chronic pain management [77].

Acupuncture is a traditional Chinese medicine therapy that targets specific points along “meridians” that run through the body. Its rationale in the treatment of endometriosis consists in its action on dysfunctional descending pain pathways [78]. However, its efficacy is mainly anecdotal and not supported by high quality evidence.

Nerve stimulation techniques have also been studied for the treatment of CS-related symptoms in women with endometriosis, although this kind of evidence is not yet applicable to clinical practice [79].

### Assessment of other COPCs and their treatment

An important aspect in the management of CS is the diagnosis and treatment, when needed, of all co-existing COPCs. In fact, it has been proven that patients with multiple COPCs often respond less robustly to treatments which are focused on one individual COPC, leaving the other COPCs not treated [51].

Adopting simple screening measures to uncover possible co-morbid COPCs is feasible in clinical practice and may facilitate referral to an appropriate specialist. These include the Rome criteria for irritable bowel syndrome (IBS) 80; the Pain, Urgency, and Frequency (PUF) score for interstitial cystitis 81; the 2011 Fibromyagia Symptom Survey for fibromyalgia [62] and should be used for screening purposes only. These conditions are in fact often diagnoses of exclusion and as such should be carried out by specialists [51].

### Treatment of myofascial pain

The recognition of a myofascial component of pain is possible through the palpation of pelvic floor muscles, typically accessed through the vagina. Although trigger points can be visualized on ultrasound and magnetic resonance imaging, imaging is not necessary for diagnosis [54].

Physical therapy for the treatment of myofascial pain may incorporate manual therapy, biofeedback, trigger point injection, pain education and cognitive behavioral strategies [14, 51].

As well as in-office physical therapy, patients are often taught home exercises, which may be cost-effective and may increase compliance. Exercises include stretching practices and massages of external and internal trigger points. The latter may be self-delivered as a home exercise using an internal wand. A retrospective study on 75 women with chronic pain reported a significant improvement in pain following transvaginal physical therapy in as many as 63% of patients. The improvement in pain was proportional to the number of sessions attended [82].

Biofeedback is an instrument-based learning process by which autonomic and neuromuscular activity is measured in order to provide visual or acoustic feedback. This technique is intended to promote awareness and self-control over physiological processes [83]. However, evidence regarding its efficacy in the treatment of endometriosis-related myofascial dysfunction is still limited [84, 85].

Although the exact mechanism of action is unknown, abdominal and pelvic floor injections are thought to disrupt trigger points. Two techniques may be used, the first, known as dry needling, is based on the mechanical insertion of a needle into the trigger point. The second, so-called wet needling, consists in the injection of an anesthetic solution. Injections are supposed to interrupt the pain pathway by relaxing and lengthening the muscle fiber [54].

### Psychological screening and treatment

Assessing psychological health is essential when treating people with endometriosis, especially considering that patients with chronic pain and concurrent psychological conditions report more severe pain and worse quality of life compared to individuals with chronic pain alone [51, 58].

Physicians may find addressing psychological issues, including mood disorders, a challenge they are not willing to pursue, as they feel they do not possess the right skills to do so. However basic communication skills such as using open-ended questions, actively listening, expressing empathy and acknowledging personal biases and stereotypes may represent valid aids in the collection of patients’ clinical history and in the recognition of signs of mood disorders [54]. Tools such as the Hospital Anxiety and Depression Score (HADS) [86] and the Beck Depression Inventory (BDI) [51, 87] may also prove useful for screening but not for diagnosis and are easily applicable to clinical practice.

Referral to mental health specialists should be suggested to all patients in whom mood disorders are known or suspected. It is of uttermost importance that patients understand that such a referral is an integral part of their treatment and not a confirmation of that “the pain is all in their head”. In fact, in many instances people’s pain symptoms are not taken seriously and are normalized - especially menstrual pain -, and it is known that these negative experiences lead to delayed diagnosis and increase the physical and emotional burden of the disease [51]. Again, there is evidence that CBT can improve quality of life following surgery [88] and several trials are ongoing in women who are not candidates for surgery.

Moreover, evidence regarding the positive effect of physical activity and exercise on mental health, and particularly on anxiety, depression and sleep disorders is increasing [89].

## Conclusions

According to As-Sanie and co-workers, women with endometriosis make on average seven visits to their primary health care professional before being referred to a specialist and nearly three-quarters of them receive a misdiagnosis [48]. On the basis of the evidence we have reported and summarized also in Table 1, it is arguable that those who do receive a correct diagnosis of endometriosis could still be receiving a misdiagnosis, as, in the majority of cases, only nociception is recognized and treated as a pain contributor. We hypothesize that so-called non-responders to progesterone may be the patients in whom further pain contributors such as inflammation, PS, CS, myofascial disorders and psychopathological conditions play such a relevant role that leaving them untreated represents an impediment to symptom resolution.

**Table 1:** Summary of articles analyzing pain contributors

AUTHOR, YEAR	JOURNAL	OBJECTIVE	STUDY TYPE	RESULTS
***Nociceptive pain contributors***				
Tokushige et al., 2006	Hum. Reprod.	To compare the innervation of peritoneal endometriotic tissue collected from 40 women with endometriosis with the peritoneum of 36 healthy women	Observational study	Peritoneal endometriotic lesions are innervated by sensory A-delta, sensory C, cholinergic and adrenergic nerve fibres. There were more nerve fibres identified in peritoneal endometriotic lesions than in normal peritoneum or endosalpingiosis lesions.
Gruber et al., 2021	Cells	To describe the pathogenesis of endometriosis, pain development and subfertility	Narrative review	Peritoneal endometriotic lesions show a hyperinnervation of sensory nerve fibers and a loss of sympathetic nerve fibers. An imbalance in the release of pro-inflammatory and anti-inflammatory sympathetic neurotransmitters seems to occur, resulting in neurogenic inflammation.
Anaf et al., 2011	Gynecol. Obstet. Invest.	To analyze the nerve density in deep infiltrating endometriotic nodules of the posterior vagina and in the adjacent healthy vaginal tissue	Prospective study	An increased number of nerve structures in endometriotic nodules may contribute to the severe neuropathic pain that characterizes these lesions.
Vercellini, 1997	Semin. Reprod. Endocrinol.	To describe pain in endometriosis	Narrative review	The specific characteristics of the lesions are more implicated in the genesis of pain than disease extension. Intraperitoneal implants may cause functional pain symptoms, whereas infiltrating lesions are responsible for organic-type pain.
Porpora et al., 1999	J. Am. Assoc. Gynecol. Laparosc.	To evaluate the relationship between prevalence and severity of CPP and stage, site, and type of endometriosis	Prospective observational study	Deep endometriosis, pelvic adhesions and ovarian cystic endometriosis were independent predictors of pelvic pain. It is not the size of ovarian cystic endometriosis but the association with adhesions that causes pelvic pain.
***Inflammation and peripheral sensitization***				
Taylor et al., 2021	Lancet	To provide an overview of endometriosis as a systemic disease	Narrative review	Endometriosis is a systemic inflammatory disease; proinflammatory cytokines and shifts in circulating immune cell populations create an inflammatory environment extending outside the pelvis.
Suryawanshi et al., 2014	Clin. Cancer. Res.	To provide a comprehensive analysis of immune gene expression in in endometriosis and EAOC	Case-control study	One third of patients with endometriosis revealed a tumor-like inflammation profile, suggesting that cancer-like immune signatures may develop earlier, in patients classified as clinically benign.
Zhang et al., 2018	Autoimmun. Rev.	To review the current understanding between autoimmunity and endometriosis	Narrative review	Changes in the immune response have been reported in women with endometriosis. Female and hormonal predominance, genetic polymorphisms, immunological abnormalities and chronic conditions are aspects in common with autoimmune diseases.
Lin et al., 2006	Endocrinology	To investigate the role of inflammatory cytokines, immune cells, and angiogenesis in the development of endometriosis in a mouse model	Animal study	The implantation of ectopic uterine tissue in the peritoneal cavity of a mouse model induced an inflammatory response. Neutrophils and macrophages are recruited and activated, producing VEGF and leading to angiogenesis in the ectopic tissue.
Klein et al., 1993	Am. J. Reprod. Immunol.	To assess whether resident leukocytes in endometriosis express IFN-γ mRNA and to compare this expression to that of normal endometrium	Case-control study	The overall concentration of T cells and macrophages expressing IFN-γ mRNA is significantly greater in endometriotic lesions as compared to the eutopic endometrium.
Symons et al., 2018	Trends. Mol. Med.	To provide in-depth insights into current understanding of the immunopathophysiology of endometriosis and highlight challenges and opportunities for future research	Narrative review	Immunological dysfunction facilitates the growth of endometriotic lesions and perpetuates disease symptoms. Targeting dysregulated immune pathways represents a potential avenue for novel therapeutic development.
Slabe et al., 2013	Geburtshilfe Frauenheilkd	To compare peripheral blood lymphocyte subpopulations during the menstrual cycle between women with peritoneal and ovarian endometriosis and healthy controls	Case-control study	The concentration of cytotoxic and activated lymphocytes did not fluctuate during the menstrual cycle in women with endometriosis. A marked increase in the concentration of regulatory T cells was detected in the luteal phase.
Chuang et al., 2015	Journal of Pathology	To evaluate potential mechanisms of immune dysfunction during endometriosis development analysing peritoneal macrophages of women with endometriosis	Case-control study	In endometriosis the phagocytic ability of endometriotic peritoneal macrophages is impaired.
Greaves et al., 2015	Am. J. Pathol.	To determine the role of estradiol in the regulation of the interaction between macrophages and nerves in peritoneal endometriosis using human tissues and a mouse model of endometriosis	Randomized controlled trial	Estrogens play a pivotal role in cross talk between neurons and macrophages. Estrogens act on nerve fibers to enhance the expression of CSF1 and CCL-2, recruiting macrophages to nerve fibers. They also act on macrophages to enhance expression of BDNF and NT-3, which further potentiates neurogenesis in endometriotic lesions.
Wu et al., 2017	J. Neuroinflammation	To describe the macrophage and nerve interaction in endometriosis	Narrative review	Retrograde menstruation promotes an inflammatory microenvironment, macrophage infiltration and hyperinnervation. Macrophages migrate into the endometriotic lesions. Within the lesions they secrete proteins that have neuroprotective properties, promoting the outgrowth of nerve fibers.
Giacomini et al., 2021	Int. J. Mol. Sci.	To provide an overview of the intersection between inflammation and genetics in endometriosis	Narrative review	The MAPK and the WNT/β-catenin cascades are signalling pathways that have been suggested to interfere with the establishment of endometriosis via several mechanisms, including apoptosis, migration and angiogenesis.
Lang *et al., 2014*	Exp. Neurol.	To describe the role of axon regeneration regulation during neuroinflammation	Narrative review	Axon regeneration regulators play a role in neuroinflammation.
Arnold et al., 2012	Brain. Behav. Immun.	To investigate possible pain mechanisms in patients with peritoneal endometriosis	Case-control study	The imbalance between sympathetic and sensory nerve fibres in peritoneal endometriosis might be involved in the maintenance of inflammation and pain.
Miller et al., 2015	Womens’ Health	To describe the role of alterations of pelvic innervation in women with endometriosis	Narrative review	The density of sensory C and sensory A-delta sympathetic and parasympathetic nerve fibers is increased in endometriotic lesions. There is a close histological relationship between endometriosis and mast cells, which play an important role in the pathogenesis of many types of chronic pain.
As-Sanie et al., 2019	Am. J. Obstet. Gynecol.	To review current practice, describe the barriers affecting diagnosis and treatment, and highlight research priorities for the future of endometriosis care	Narrative review	There is only a marginal relationship between number of lesions, severity of disease, symptoms, and overall impact on quality of life. Comprehensive and interdisciplinary approaches that take patients’ holistic needs into account are needed.
***Central sensitization and COPCs***				
Ren et al., 2007	Mol. Neurobiol.	To describe the role of BDNF-TrkB signaling and NMDA receptors in pain facilitation and activity-dependent plasticity in pain modulation	Narrative review	Pain modulatory circuitry in the brainstem exhibits considerable plasticity in response to injury. The synaptic plasticity observed in the pain pathways shares many similarities with other forms of synaptic plasticity.
Nijs et al., 2021	J. Clin. Med.	To povide an overview on past and present IASP criteria for nociplastic pain	Narrative review	In 2017, the IASP introduced the term “nociplastic pain” as a third mechanistic pain descriptor in addition to nociceptive and neuropathic pain, providing a label to patients having a predominant central sensitization type of pain.
Till et al., 2022	Obstet. Gynecol. Clin. North. Am.	To review individual COPCs, risk factors and common mechanisms. To review evaluation and communication strategies to establish a productive therapeutic relationship	Narrative review	Patients with co-occurring COPCs may benefit from the addition of treatments aimed at central sensitization, including pharmacologic and non-pharmacologic strategies. An interdisciplinary approach is essential, as no single provider has adequate expertise to manage all these conditions alone.
Raimondo et al., 2022	J. Minim. Invasive. Gynecol.	To assess the prevalence of CS and its association with demographic and clinical factors in patients with endometriosis	Cross-sectional study	The prevalence of CS was 41.4% among patients with endometriosis. Moderate-to-severe pain symptoms were significantly more frequent in the CS group, except for dyschezia.
Orr et al., 2022	Pain	To identify a CSI cut-off in the endometriosis population to discriminate individuals with significant central pain contributors	Cross-sectional study	A CSI score > 40 may identify patients with endometriosis with pain contributors related to CS. A significant correlation between an increasing score of CSS and increasing pain scores was observed.
Marchand, 2008	Rheum. Dis. Clin. North. Am.	To describe the physiology of pain mechanisms	Narrative review	Keeping in mind the heterogeneity of pain responses and the unique characteristics of individual patients leads to better patient care. A greater understanding of the neurophysiologic mechanisms underlying the development and maintenance of pain could prove useful to reinforce inhibitory mechanisms or reduce the hyperactivity of the nociceptive response.
Ezra et al., 2019	Front Psychiatry	To describe the Four-Cluster Spectrum of Mind-Body Interrelationships	Narrative review	Diseases may be classified in four clusters in which the mind-body ratio is progressively increasing (cluster 1: organic conditions; cluster 2: stress-exacerbated, typically inflammatory diseases; cluster 3: functional somatic syndromes; cluster 4: conversion disorders).
Morotti et al., 2017	Eur. J. Obstet. Gynecol. Reprod. Biol.	To describe mechanism of pain in endometriosis	Narrative review	Endometriosis-associated pain is similar to that of other chronic pain conditions in its engagement and alteration of the CNS.
Green et al., 2022	Clin. Obstet. Gynecol.	To provide a systematic approach to persistent pain in patients with endometriosis	Narrative review	Treatment of chronic pain is best achieved by addressing both peripheral and central components of pain. A combination of mind-body and interdisciplinary interventions is recommended as well as surgery and single-agent pharmacotherapy.
***Myofascial contributors***				
Ross et al., 2021	J. Midwifery Womens Health	To describe the presence of myofascial pelvic pain in chronic pelvic pain syndromes	Narrative review	Myofascial pain may be the missing piece when conventional treatments fail to completely relieve patient’s discomfort. Several myofascial pain therapies are low-intervention and significantly enhance patients’ quality of life.
Aredo et al., 2017	Semin. Reprod. Med.	To provide a background to understand how endometriosis facilitates remodeling of neural networks, contributing to sensitization and to the generation of MTrPs	Narrative review	Over time, CS creates a process for pain sustention that is independent of the initial pathology and is potentially reversible. Viscerosomatic convergence may not only provide the means for pain referral to somatic structures but also govern the reflex that induces muscle spasm and the formation of MTrPs.
Phan et al., 2021	Eur. J. Pain.	To characterize the presence and distribution of pain, myofascial dysfunction and sensitisation beyond the pelvis in women with endometriosis-associated chronic pelvic pain	Cross-sectional study	Women with endometriosis-related chronic pelvic pain may frequently present with myofascial dysfunction and sensitization outside of the pelvic area, which could be caused or sustained by persistent pelvic floor spasm.
***Psychological contributors***				
Koller et al., 2023	JAMA NEtw. Open	To investigate whether pleiotropy contributes to the association of endometriosis with depression, anxiety, and eating disorders	Retrospective study	Endometriosis affects women’s mental health through pleiotropic processes. There is genetic and phenotypic proof of the mechanisms underlying the psychiatric comorbidities of endometriosis.
Chen et al., 2016	J. Affect. Disord.	To investigate the temporal association between endometriosis and depression or anxiety disorders	Longitudinal study	Compared to women without endometriosis, affected women are more likely to experience major depressive disorders and anxiety disorders in later life.
Maulitz et al., 2022	Front. Neuroendocrinol.	To analyze studies reporting comorbid mental disorders in endometriosis based on the ICD/DSM criteria in the context of available neuroimaging studies	Narrative review	Depression, anxiety, bipolar disorder, alcohol/drug dependency and hyperactivity disorder are all more prevalent in women with endometriosis. This may be explained by pain, presence of comorbidities, inflammation, hormonal treatment, genetic predisposition.
Doney et al., 2022	Eur. J. Neurosci.	To provide an overview of inflammation-related mechanisms involved in mood regulation and stress responses on animal models.	Narrative review	Blood-brain and gut barriers are made more brittle and hyperpermeable by stress-induced, exaggerated inflammation. This may be brought on by dysbiosis, an imbalance in microbial populations and changes to the gut-brain axis, which is crucial for the synthesis of the mood-regulating neurotransmitter serotonin.

**List of abbreviations**:

CPP = chronic pelvic pain; EAOC = endometriosis-associated ovarian cancer; VEGF = vascular endothelial growth factor; IFN-γ mRNA = interferon gamma messenger RNA; CSF1 = colony stimulating factor 1; CCL-2 = chemokine ( C-C motif) ligand 2; BDNF = brain derived neurotrophic factor; NT-3 = neurotrophin-3; MAPK = mitogen activated protein kinase; WNT = Wingless and Int-1; NMDA = N-methyl-D-aspartate; IASP = International Association for the Study of Pain; COPCs = chronic overlapping pain conditions; CS = central sensitization; CSI = central sensitization inventory; CSS = central sensitivity syndromes; CNS = central nervous system; MTrPs = myofascial trigger points; ICD = International Classification of Diseases; DSM = Diagnostic and Statistical Manual of Mental Disorders

Further research is certainly necessary not only to confirm such hypothesis but also to identify an effective treatment for each pain contributor. Meanwhile, providing patients with a clear overview of all the different treatments they could benefit from and building realistic expectations on what such treatments entail and how they may prove useful, may represent a starting point. Diet, physical exercise, physical therapy and psychotherapy may not be sufficient to resolve patients’ symptoms but may certainly be necessary, in addition to hormonal therapy, to address the multiple pathogenic facets of endometriosis.

## Data Availability

Not applicable.

## Abbreviations

COCs: Combined oral contraceptives
PR-A: Progesterone receptors A
PR-B: Progesterone receptors B
ERβ: Estrogen receptorsβ
PS: Peripheral sensitization
CS: Central sensitization
PF: Peritoneal fluid
IL-1β: Interleukin 1β
IL-6: Interleukin 6
IL-8: Interleukin 8
NGF: Nerve growth factor
COPCs: Chronic overlapping pain conditions
OR: Odds ratio
NSAIDs: Non-steroidal anti-inflammatory drugs
FODMAP: Fermentable oligo-, di-, monosaccharides, and polyols
CSI: Central Sensitization Inventory
TCAs: Tricyclic antidepressants
SNRIs: Serotonin–norepinephrine reuptake inhibitors
CBT: Cognitive behavioral therapy
IBS: Irritable bowel syndrome
PUF: Pain, Urgency, and Frequency
HADS: Hospital Anxiety and Depression Score
BDI: Beck Depression Inventory
